# Supportive Care and Symptom Management for Patients With Immunoglobulin Light Chain (AL) Amyloidosis

**DOI:** 10.3389/fonc.2022.907584

**Published:** 2022-06-23

**Authors:** Christopher E. Jensen, Mirnela Byku, Gerald A. Hladik, Koyal Jain, Rebecca E. Traub, Sascha A. Tuchman

**Affiliations:** ^1^ Division of Hematology, University of North Carolina School of Medicine, Chapel Hill, NC, United States; ^2^ Cecil G. Sheps Center for Health Services Research, University of North Carolina, Chapel Hill, NC, United States; ^3^ Division of Cardiology, University of North Carolina School of Medicine, Chapel Hill, NC, United States; ^4^ Division of Nephrology and Hypertension, University of North Carolina School of Medicine, Chapel Hill, NC, United States; ^5^ Department of Neurology, University of North Carolina School of Medicine, Chapel Hill, NC, United States; ^6^ Lineberger Comprehensive Cancer Center, University of North Carolina, Chapel Hill, NC, United States

**Keywords:** AL amyloidosis, supportive care, symptom management, nephrotic syndrome, cardiac amyloidosis, neuropathy

## Abstract

Immunoglobulin light chain (AL) amyloidosis is a disorder of clonal plasma cells characterized by deposition of amyloid fibrils in a variety of tissues, leading to end-organ injury. Renal or cardiac involvement is most common, though any organ outside the central nervous system can develop amyloid deposition, and symptomatic presentations may consequently vary. The variability and subtlety of initial clinical presentations may contribute to delayed diagnoses, and organ involvement is often quite advanced and symptomatic by the time a diagnosis is established. Additionally, while organ function can improve with plasma-cell-directed therapy, such improvement lags behind hematologic response. Consequently, highly effective supportive care, including symptom management, is essential to improve quality of life and to maximize both tolerance of therapy and likelihood of survival. Considering the systemic nature of the disease, close collaboration between clinicians is essential for effective management.

## Introduction

Immunoglobulin light chain (AL) amyloidosis is an acquired disorder of clonal plasma cells characterized by the production of light chains which are predisposed to misfolding and subsequent aggregation into ordered amyloid fibrils (1). These fibrils deposit in a variety of tissues, leading to end-organ injury. Precise mechanisms of end-organ injury are incompletely characterized, though both tissue structural disruption and cytotoxicity secondary to cellular internalization of amyloid fibrils and amyloidogenic precursors appear to contribute (2–4). The propensity of amyloid fibrils to deposit in various organs varies. The kidneys and heart are most commonly affected, though any organ outside the central nervous system can be involved (1, 5, 6).

Estimates of the incidence of AL amyloidosis have been variable given the lack of large population-based databases; however, case series and claims analyses from the United States have yielded an estimated incidence of approximately 1 per 100,000 persons per year (1, 7–9). AL amyloidosis is more common with advancing age, with peak incidence in the seventh to eighth decade of life (7–9).

**Figure 1:**
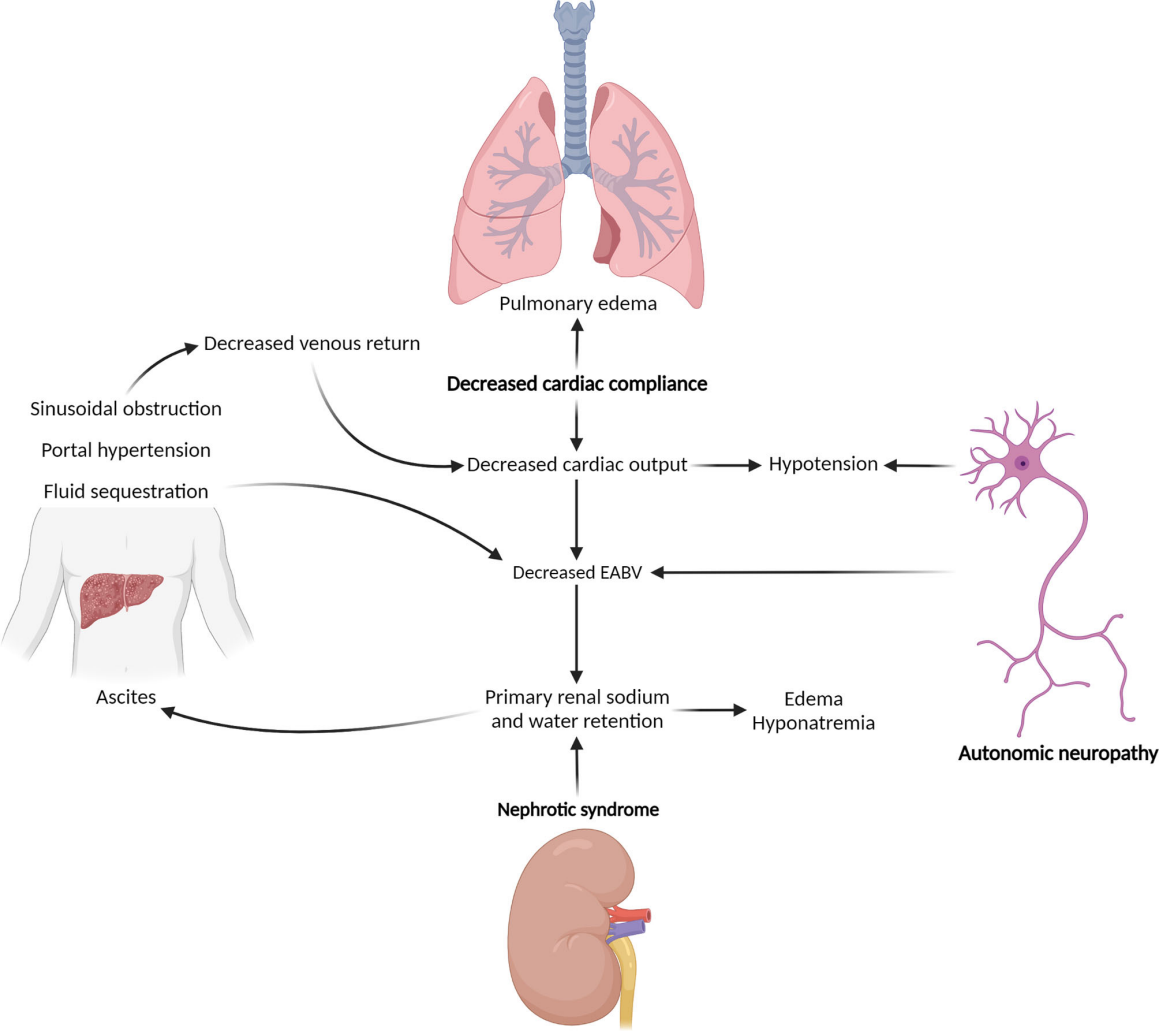
Hemodynamic effects of end-organ injury related to AL amyloidosis. Management of hypotension and third-spacing of fluid in the setting of AL amyloidosis is complicated by pathophysiologic feedback loops, by which injuries to different organ systems compound one another. Bold type indicates primary effects. EABV, effective arterial blood volume. (Created with BioRender.com).

The backbone of therapy is directed at the underlying plasma cell clone. However, diagnosis is often delayed, and improvement in organ function typically lags behind hematologic response (10, 11). Effective symptom management and supportive care are therefore essential, both to improve quality of life for its own sake and to maximize tolerance of plasma-cell-directed therapy (11, 12). Involvement of different organ systems can result in symptoms that compound one another (**Figure 1**), and plasma-cell-directed therapy may exacerbate disease-related symptoms, making supportive management challenging. Patients are particularly vulnerable to deterioration in their quality of life shortly after initiation of therapy (13). In these complex circumstances, patients benefit from multidisciplinary co-management.

## Clinical Case

A 62-year-old man with a history of hypertension developed progressive fatigue, constipation, nausea, and anorexia resulting in approximately 30 pounds of unintentional weight loss over the course of 6 months, followed by new onset dizziness on standing from a seated position. Blood work obtained by his primary care provider was notable for elevation in serum alkaline phosphatase to >1,000 U/L (upper limit of normal 126) and hypoalbuminemia to 2.3 g/dL (reference 3.5-5.0), prompting referral to gastroenterology. An abdominal MRI demonstrated heterogenous enhancement in the liver but normal liver span. Hepatic biopsy was obtained, revealing extensive sinusoidal amyloid deposition with associated hepatic plate atrophy. Amyloid fibril typing by liquid chromatography tandem mass spectrometry was consistent with AL amyloidosis. He was started on midodrine for orthostatic hypotension and was referred to hematology.

Initial staging included serum protein electrophoresis with a monoclonal spike (M-spike) of 0.5 g/dL, typed as lambda free light chains *via* immunofixation. Kappa free light chain (FLC) concentration was 1.62 mg/dL (reference 0.33-1.94), with lambda FLCs of 39.98 mg/dL (reference 0.57-2.63), FLC ratio 0.04 (reference 0.26 - 1.65), and differential FLCs 38.36 mg/dL. Blood counts were normal. Bone marrow biopsy demonstrated a normocellular marrow with 18% plasma cell infiltrate. Karyotype was normal, though a plasma cell proliferation fluorescence *in-situ* hybridization panel demonstrated a hyperdiploid clone harboring a 1q duplication. Skeletal imaging was negative. Estimated glomerular filtration rate was >90 ml/min per 1.73 m^2^, though proteinuria on 24-hour urine collection was 3.4 grams and serum albumin was 2.4 g/dL, compatible with presumed renal amyloidosis. Troponin T was 0.068 mcg/L (ULN 0.015), and N-terminal pronatriuretic peptide type-B (NT-proBNP) was 1,966 ng/L (ULN 82). Transthoracic echocardiogram showed severely increased left ventricular wall thickness and severely abnormal global longitudinal strain, compatible with cardiac amyloidosis. A final diagnosis of AL amyloidosis with hepatic, renal (stage I), cardiac (Mayo 2012 stage IV), and probable autonomic neuropathic involvement was established (14, 15).

Before therapy could be initiated, the patient experienced worsening anorexia, nausea, and constipation with associated functional decline and worsening orthostatic hypotension, prompting inpatient admission for gentle intravenous fluids, aggressive antiemetic and bowel regimen titration, and initiation of systemic therapy under close monitoring. In the context of his end-organ dysfunction, he was treated with dose-attenuated therapy with weekly bortezomib 0.7 mg/m^2^, cyclophosphamide 300 mg/m^2^, and dexamethasone 12 mg. His treatment course was complicated by the development of ascites requiring serial therapeutic paracentesis, marked hyponatremia requiring treatment with urea, and worsening, midodrine-refractory orthostatic hypotension prompting initiation of droxidopa. Despite escalation of his plasma-cell directed therapy to full, per-protocol dosing as tolerated (16), he experienced no hematologic response with rising serum cardiac biomarkers (17).

He was transitioned to subcutaneous daratumumab as second-line systemic therapy, with treatment complicated by persistent anasarca/ascites and development of chronic diarrhea. He also experienced recurrent syncope thought to be secondary to an intersection of autonomic-neuropathy-related orthostatic hypotension, intravascular volume depletion from diarrhea and anorexia, third-spacing of fluid related to ascites accumulation due to sinusoidal obstruction and portal hypertension, hypoalbuminemia, and diastolic heart failure. He underwent placement of a peritoneovenous shunt placement, which obviated the need for paracentesis. In addition, the severity of hypotension diminished presumably due to increased venous return. He was continued on droxidopa and initiated on twice-weekly albumin infusions with improvement in hypotension and syncope. He achieved a very good partial response (VGPR) according to AL response criteria following 6 cycles of daratumumab, with subsequent improvement in serum cardiac biomarkers (17). He was maintained on daratumumab and supportive interventions as above thereafter, with gradual, further improvement in organ function and, consequently, overall functional status.

## Supportive Care by Organ System in AL Amyloidosis

### Kidney

Kidney involvement is the most frequent end-organ manifestation of AL amyloidosis, with prevalence estimates varying by series and ranging from just above 50% to as high as 80% (5, 6). Kidney deposition most commonly involves the glomerular mesangium and basement membrane, with resultant proteinuria as a prominent clinical feature (18). Proteinuria of greater than 3 grams per 24 hours has been described in the majority of patients with renal amyloidosis at the time of diagnosis (5, 19). Impairment in glomerular filtration rate is less common, with a reported prevalence of up to 50% among all patients with AL amyloidosis, while progression to end-stage renal disease occurs in approximately 15-30% at some point over the disease course (14, 20, 21). Plasma-cell directed therapy remains the cornerstone of amyloid-associated kidney disease, and kidney involvement may be particularly responsive to systemic therapy compared to other end-organ involvement (22, 23).

While kidney involvement is common, clinical manifestations of renal amyloid deposition vary widely from asymptomatic proteinuria to nephrotic syndrome, with associated volume overload, peripheral edema, and ascites, despite intravascular hypovolemia. The presence of ascites should prompt a thorough evaluation for hepatic involvement and portal hypertension. Supportive care is largely directed toward those with symptomatic manifestations. Non-pharmacologic management may include compression stockings and leg elevation. For individuals in whom these measures are insufficient, treatment relies on the use of loop diuretics, particular long-acting loop diuretics, along with salt and fluid restriction in patients with evidence of extracellular fluid expansion and sodium overload. Intermittent albumin infusions may be beneficial in select patients with severe nephrotic syndrome when hypotension limits the ability to achieve euvolemia with diuretics, particularly if heart failure is also present (24).

As in other proteinuric conditions, renin-angiotensin-aldosterone system blockade (with either angiotensin-converting enzyme inhibitors or angiotensin receptor blockers) may minimize progression of proteinuria, though evidence is extrapolated from other disease states (25). Moreover, these agents must be used with caution, as they can exacerbate hypotension due to amyloid-mediated cardiomyopathy or autonomic neuropathy. Similarly, although AL carries with it a risk of venous thromboembolic disease, particularly in the setting of severe hypoalbuminemia (< 2.5 g/dL), utilization of prophylactic anticoagulation, as extrapolated from other etiologies of nephrotic syndrome, must be balanced against the prevalence of bleeding diatheses due to AL amyloidosis (26). Consideration may also be given to kidney transplantation for those with advanced amyloid-related chronic kidney disease, particularly among those who achieve a deep hematologic response, given evidence of long-term patient and graft survival in such individuals (23).

### Cardiac

Amyloid cardiomyopathy is the second most common end-organ manifestation of AL amyloidosis following renal involvement, with overt heart failure developing in 30-40% of patients with AL amyloidosis over the course of their disease trajectory (5, 6). Subclinical cardiomyopathy, detected by MRI or echocardiography, low voltages on electrocardiogram, and elevated serum cardiac biomarkers, sometimes precedes overt heart failure symptoms (27). Echocardiography typically demonstrates increased left and right ventricular wall thickness, interatrial septal thickening, diastolic dysfunction with increased filling pressures, and an abnormal pattern of longitudinal myocardial strain that relatively spares the left ventricular apex, as in the preceding clinical case (24). Discordance between hypertrophy observed on echocardiography and low voltage observed on electrocardiogram is a particularly specific finding for cardiac amyloid involvement.

Cardiac involvement is a major determinant of prognosis and tolerance of plasma-cell-directed therapy (11, 24, 28). Severity of cardiac involvement, by serum biomarkers and clinical heart failure, should consequently be factored into initial treatment strategy. For instance, steroids can contribute to worsening edema or third-spacing, and bortezomib may be uncommonly associated with arrythmias (29). Individuals with limited performance status (Eastern Cooperative Oncology Group Performance Status 4), advanced clinical heart failure (New York Heart Association class III or IV), or higher stage cardiac biomarkers (Mayo 2012 stage III to IV) who are otherwise candidates for systemic treatment should initially be managed with intensity-attenuated therapy. Strategies include dose-reduction of a standard regimen (as in the preceding case) or withholding one agent of a triplet regimen, with subsequent intensification of therapy if an initial cycle is well-tolerated (11).

Supportive therapy for amyloid cardiomyopathy focuses on maintenance of appropriate volume status *via* salt and water restriction and use of diuretics. Dual therapy with a loop diuretic (furosemide, torsemide, bumetanide) and aldosterone antagonist (typically spironolactone) may be utilized (27). Caution must be exercised to avoid over-diuresis, as volume depletion may exacerbate renal dysfunction in those with amyloid-related renal disease and orthostatic hypotension in those with autonomic neuropathy (24). Neurohormonal blockading agents, including angiotensin-converting enzyme inhibitors, angiotensin receptor blockers, and beta blockers, are often poorly tolerated in patients with amyloid cardiomyopathy secondary to hypotension and offer no known mortality or morbidity benefit in this cohort (27). Patients often feel better with withdrawal of neurohormonal blocking therapy. As with renal transplantation, heart transplant can be considered for those with advanced heart failure secondary to amyloidosis and limited extra-cardiac organ involvement by AL. Outcomes with transplant are again more favorable among those with deep hematologic responses (30, 31).

Atrial arrythmias are common in individuals with cardiac amyloid deposition (32, 33). Rate control can be challenging in this setting, as beta blockers or non-dihydropyridine calcium channel blockers are poorly tolerated due to concurrent myocardial dysfunction. Digoxin has been historically viewed as contraindicated in the setting of cardiac amyloidosis, given prior evidence of binding of digoxin to amyloid fibrils and subsequent excess cardiac toxicity, though more recent evidence suggests that it may be used sparingly and cautiously (34). Additionally, direct current cardioversion can result in myocardial stunning with significant bradycardia, conduction block and even asystolic arrest, especially in patients on rate-controlling agents. Consequently, amiodarone may be the best tolerated option for management of arrythmia (27), though care must be taken to minimize interactions with QT prolonging medications (e.g. antiemetics) and those transported by p-glycoprotein. Individuals with amyloid-related arrhythmias also appear to be particularly vulnerable to formation of intracardiac thrombi compared to those with arrythmias in the absence of amyloidosis, though therapeutic anticoagulation must be carefully monitored particularly in patients with amyloid-related coagulopathy (35). Direct oral anticoagulants (DOACs) may be utilized in this setting, with some evidence for superior outcomes when compared to vitamin K antagonists (36).

Patients with amyloidosis are also at an increased risk for atrioventricular block resulting in significant bradycardia and cardiac arrest. Syncope in these patients should be investigated promptly as it is a poor prognostic marker. Pacemaker placement is indicated in such patients. Defibrillator placement can be considered in those with otherwise acceptable anticipated survival and concomitant systolic dysfunction with ejection fraction less than 35% and/or evidence of ventricular arrythmia on cardiac monitoring (37). However, patients with cardiac amyloidosis appear to have inferior outcomes following defibrillator placement compared to individuals with other nonischemic cardiomyopathies, and placement does not appear to improve survival significantly (38–40).

As in other etiologies of heart failure, iron deficiency is common among individuals with cardiac amyloidosis, with a recent study describing a prevalence of 45% among individuals diagnosed with cardiac AL amyloidosis (41). While the impact of iron repletion has been little studied in this subpopulation, data from individuals with other etiologies of heart failure support intravenous iron supplementation for individuals with cardiac AL amyloidosis, in the absence of concurrent contraindications (42, 43).

### Neuropathy

Neuropathy is present in approximately 20-30% of individuals with AL amyloidosis (5, 44). The most common neurologic manifestation is a polyneuropathy characterized by a length-dependent, sensory-predominant impairment resulting in numbness, paresthesias, or pain (10). Motor manifestations are less common but may occur as well. These symptoms are typically progressive and may be subtle initially. Consequently, diagnosis is often delayed if neuropathy is the initial symptomatic manifestation of AL (44). Additionally, while bortezomib-based first-line treatment regimens have resulted in substantially improved hematologic and end-organ response rates, bortezomib may induce or exacerbate peripheral neuropathy (45–47). Fortunately, while neurologic complications of AL amyloidosis were initially described as irreversible, more recent evidence indicates that both sensorimotor and autonomic neuropathy may improve in a minority of cases following plasma-cell-directed therapy (6, 48–51). More broadly, systemic therapy can prevent symptoms of neuropathy from worsening. On the other hand, should a patient experience worsening symptoms while receiving bortezomib-based therapy, consideration must be given to bortezomib dose-reduction or discontinuation.

Recommendations regarding symptomatic therapies for AL sensorimotor neuropathy are largely extrapolated from other settings, particularly diabetic neuropathy. However, the evidence base for the comparative effectiveness of pharmacologic agents even in the setting of these more common neuropathies is limited by a lack of direct comparisons and relatively short term follow up in studies (52, 53). A trial of gabapentinoids (gabapentin, pregabalin) or serotonin-norepinephrine reuptake inhibitors (venlafaxine, duloxetine) is appropriate. If these are ineffective, later line agents include carbamazepine, topiramate, lamotrigine as well as topical lidocaine or cannabinoids. Tricyclic antidepressants may be less favored in the context of AL neuropathy due to their risk of exacerbating orthostatic hypotension, particularly among those with concurrent autonomic neuropathy (54, 55).

Autonomic neuropathy is a less common manifestation of peripheral nervous injury from AL amyloidosis, with a prevalence of approximately 15% (5, 51). Autonomic neuropathy typically manifests as orthostatic hypotension, though other symptoms such as gastrointestinal motility disorders, urinary dysfunction, or erectile dysfunction may also occur (51). Orthostatic hypotension may be particularly challenging to manage given the frequency of concurrent renal and cardiac involvement, with associated compounding hemodynamic derangements (**Figure 1**). Initial treatment should focus on discontinuation of exacerbating medications, as able, and use of fitted elastic compression stockings to support blood pressure (56).

For patients with hypotension not adequately managed with non-pharmacologic measures, the oral sympathomimetic midodrine may utilized (57). Potential adverse effects include anxiety, depression, gastrointestinal upset, urinary retention and, perhaps most problematically, supine hypertension (58). Alternatively, droxidopa, an oral prodrug of norepinephrine, is a newer sympathomimetic agent also approved for symptomatic orthostatic hypotension. Droxidopa appears to have a lower risk of supine hypertension compared to midodrine, with nausea and headache as the most frequently described adverse effects (59, 60). While data again comes from the setting of other disease states, droxidopa has also been utilized successfully in the setting of AL amyloidosis (61, 62).

Pyridostigmine, an acetylcholinesterase inhibitor, may also be used for management of orthostatic hypotension (63). The primary data supporting this approach derives from a trial in which pyridostigmine was utilized in combination with midodrine, and when used as monotherapy, pyridostigmine appears to less efficacious than midodrine (64, 65). Pyridostigmine may have additional symptomatic benefits in those with autonomic neuropathy *via* improvement in urinary retention or constipation related to gastrointestinal dysmotility. Fludrocortisone has also been utilized for treatment of orthostatic hypotension; however, its use may exacerbate volume overload in the context of concurrent renal disease or cardiomyopathy (63).

Upper extremity neurologic symptoms related to carpal tunnel syndrome may also be present. This syndrome is described in approximately 15-20% of individuals with AL amyloidosis (5, 66). Carpal tunnel syndrome is a phenomenon of soft-tissue deposition of amyloid with secondary compression of the median nerve, rather than neurologic involvement of amyloid primarily. Consequently, carpal tunnel syndrome requires separate management from the neuropathic symptoms above. Interventions can include appropriate bracing, though surgical carpal tunnel release may also be required (63).

Similar neural compressive processes may rarely occur at other sites, though such manifestations are rare (67). Among these, spinal stenosis secondary to expansion of the ligamentum flavum is of particular note, as it may drive extremity numbness, weakness, and neuropathic pain which mimic the far more common amyloid peripheral neuropathy.

### Pulmonary

Clinically significant pulmonary involvement with systemic amyloidosis is uncommon compared to asymptomatic amyloid deposition in lung vasculature, and overt respiratory symptoms of a primary pulmonary etiology are infrequent in the absence of advanced cardiac involvement (68). Pulmonary involvement with systemic amyloidosis may manifest as either alveolar-septal deposition (with associated impaired gas exchange) or pleural deposition (manifesting as recurrent pleural effusions) (68, 69). These patterns of deposition must be distinguished from isolated nodular amyloid deposits in the lung, which are generally not associated with systemic AL amyloidosis, affect only the lungs, and are instead related to other, typically indolent, B-cell clonal processes (69, 70). Additionally, pulmonary symptoms may arise as a result of therapy rather than amyloid deposition, as daratumumab has been associated with cough (with or without associated respiratory tract infection) (71, 72).

There is limited specific data regarding changes in pulmonary function following plasma-cell directed therapy, and there are few specific supportive interventions for alveolar-septal disease other than volume optimization and supplemental oxygen as needed (69). Pleural effusions may be addressed *via* thoracentesis, though effusions will typically recur following drainage, and pleurodesis can be considered if repeated thoracenteses are required (68).

### Gastrointestinal and Hepatic

Gastrointestinal (GI) endothelial deposition of AL amyloid is relatively infrequent, with a reported prevalence of less than 10% of cases (73–75). Associated intestinal malabsorption may contribute to both weight loss and diarrhea. However, motility disorders including gastroparesis and altered colonic transit time (resulting in diarrhea or constipation) are more likely to be related to autonomic dysfunction rather than direct involvement of the GI endothelium (76, 77). Diagnosis may be challenging given non-specific symptoms, including dysphagia, weight loss, abdominal pain, nausea, and vomiting (77), as well as the inability of luminal GI biopsies to detect amyloid present in autonomic nerves. Once a patient is on therapy, as in the case of amyloid-related neuropathy, bortezomib may induce or exacerbate bowel dysfunction, contributing to either constipation or diarrhea (45, 71). Here again, bortezomib dose-modification should be considered if it is implicated in exacerbating a patient’s symptoms.

Otherwise, supportive care depends on the individual patient’s symptoms, though symptoms are unfortunately often poorly responsive to pharmacologic interventions (77). Metoclopramide or laxatives may be trialed for slow GI motility, while loperamide and diphenoxylate-atropine may be utilized for diarrhea. For diarrhea related to malabsorption, octreotide may provide additional benefit.

Hepatic involvement with AL amyloid may diagnosed in the context of serum alkaline phosphatase elevation, along with either liver biopsy demonstrating interstitial deposition of amyloid fibrils or hepatomegaly in the absence of heart failure (77) (Serum alkaline phosphatase elevations may result from either hepatic amyloid deposition or indirectly due to congestive hepatopathy.) Hepatic AL may result in right upper quadrant pain, early satiety, and weight loss, particularly if concurrent splenomegaly is present. An enlarged, hard, nodular liver edge may be palpated on physical exam.

Sinusoidal hepatic obstruction can lead to portal hypertension, ascites accumulation, and need for diuretic therapy, paracentesis, or peritoneovenous shunting. Transjugular intrahepatic portosystemic shunts are often contraindicated because of diastolic heart failure. In such cases, a peritoneovenous shunt (Denver shunt) can be considered after consultation with hepatology and cardiology. These shunts should be placed by an experienced interventional radiologist. Contraindications include decompensated heart failure, advanced chronic kidney disease, variceal bleeding, loculated ascites, active infection, liver failure, or bloody ascites. Complications include disseminated intravascular coagulation (0-5%), pulmonary edema (0-5%), and pulmonary embolus (0-5%) (78). Ultimately, management of hepatic AL largely depends on systemic therapy and hematologic response, which can result in improvement in hepatic synthetic function and reabsorption of deposited amyloid (77).

### Macroglossia

Enlargement of the tongue (macroglossia) with scalloped dental indentations is classically described as a feature of amyloidosis, though this finding is only present in about 10-20% of cases (5, 66, 79). Secondary effects of macroglossia include speech difficulties, jaw malocclusion, dysphagia, obstructive sleep apnea, and, if severe, more persistent airway obstruction and dysphagia (76). Unfortunately, even if a hematologic response is achieved with systemic therapy, macroglossia often does not regress (80). Radiation therapy appears to have little efficacy in this setting (81). Surgical approaches for tongue debulking have been described that can result in some symptomatic improvement in severe cases; however, procedural morbidity is high and results are inconsistent (82, 83).

### Sicca Syndrome

Amyloid fibrils may also deposit in salivary glands, and amyloid deposits may be found in a majority to patients with AL amyloidosis if minor salivary gland biopsy is pursued (84, 85). Substantial deposition resulting in impaired major salivary gland function mirroring Sjögren syndrome is far less frequent, though this has also been described (86, 87). Associated symptoms include dry mouth and consequent dental decay. Given the rarity of this condition, management must largely be extrapolated from the setting of Sjögren syndrome, with consideration of non-pharmacologic salivary stimulants (sugar-free candies, lozenges) for mild symptoms, pharmacologic stimulants (pilocarpine, cevimeline) for moderate symptoms, and artificial saliva substitutes for patients with severe symptoms due to absent residual salivary gland function (88).

### Other Soft Tissue Deposition

Rare soft tissue and skeletal deposition at other sites has also been described, including skin nodules, myopathy, and periarticular deposition resulting in arthropathy (66). Skeletal muscle involvement can present with weakness, myalgia, and atrophy or pseudohypertrophy (89). Concurrent neuropathy is often present, though early or prominent proximal muscle weakness may point to presence of myopathy. Elevated serum creatine kinase (CK) also suggests the presence of myopathy, though CK levels may be normal in as many as half of cases (89). Myopathic findings on electromyography are more specific, though muscle biopsy may be indicated if the clinical picture is unclear. Therapy is largely directed at the underlying plasma cell clone (89).

Joint involvement is most commonly multifocal, characterized by a nonerosive polyarthritis; however, oligo- or monoarthritis may also occur (90). Treatment with nonsteroidal anti-inflammatory drugs (NSAIDs) and systemic or intraarticular corticosteroids generally yields some symptomatic improvement; however, caution must be exercised with the use of high doses or prolonged courses of NSAIDs in the context of concurrent renal and/or GI disease, or amyloid coagulopathy (90, 91). Symptomatic improvement has also been reported in the majority of patients with amyloid arthropathy and concurrent multiple myeloma who receive plasma-cell-directed therapy (90).

### Coagulopathy

In additional to gastrointestinal bleeding as above, a more generalized bleeding diathesis may be observed in 10-25% of patients (92, 93). Acquired factor X deficiency has been implicated in prior retrospective studies (92–94). However, pathological bleeding has also been described in patients with amyloidosis without abnormalities on routine coagulation testing (95). Additionally, not all patients with abnormal coagulation studies exhibit a clinically overt bleeding diathesis (92). Bleeding tendencies may consequently be multifactorial, involving hemostatic deficits, hyperfibrinolysis, and localized amyloid deposition (as in the GI tract) (96). Therapy should be directed at the underlying predominant cause of bleeding for the individual patient, if identified, though more generalized measures such as the administration of recombinant activated factor VII may be required in cases of severe or periprocedural bleeding (96). Like other manifestations of AL, coagulopathy also tends to improve with successful treatment of the underlying AL.

### Endocrinopathy

AL amyloidosis may be linked to higher rates of thyroid dysfunction, an association most comprehensively assessed in the Mayo Clinic experience (97). This series described a 19% rate of thyroid function abnormalities among individuals with newly diagnosed AL amyloidosis. Most of these individuals (12% of cohort) had subclinical hypothyroidism (elevated thyroid-stimulating hormone levels with normal free thyroxine levels), though the presence of hypothyroidism was nevertheless independently associated with inferior survival. Consequently, routine assessment of thyroid function in individuals with newly diagnosed AL amyloidosis is appropriate. Additionally, thyroid function should be considered in those receiving amiodarone as above, as this medication is associated with increased rates of thyroid dysfunction.

### Infection

In the context of immunosuppressive plasma-cell directed therapies, patients treated for AL amyloidosis are at increased risk of infection; however, a role for antibacterial prophylaxis has not been established. Herpes simplex virus (HSV) prophylaxis should be administered for those receiving bortezomib or daratumumab-containing regimens (98). *Pneumocystis jirovecii* pneumonia (PJP) prophylaxis can be considered, though its use is less clearly mandated than HSV prophylaxis. For instance, recent investigational protocols have deferred to local institutional standards regarding PJP coverage (99).

## Conclusion

Given the systemic nature of AL amyloidosis along with the inherent delay between initiation of therapy and end-organ response, effective supportive care is a critical component of overall management. Such care should aim both to improve a patient’s quality of life and to maximize tolerance of systemic therapy. Additionally, the intersecting and compounding effects of amyloid deposition in individual end-organs make supportive management challenging, and this scenario may be further complicated by treatment toxicity. As such, a supportive care regimen must be reevaluated and adjusted throughout an individual’s treatment course. Multidisciplinary care is essential to optimizing outcomes, and patients may benefit from treatment at a center with experience in management of this complex disorder.

## Author Contributions

CJ and ST conceived the concept. CJ and GH designed the figures. CJ wrote the manuscript, and the other authors listed made substantial, direct intellectual contributions to the work and approved it for publication.

## Funding

This work was partially supported by a National Research Service Award Post-Doctoral Fellowship from the Agency for Healthcare Research and Quality sponsored by The Cecil G. Sheps Center for Health Services Research, The University of North Carolina at Chapel Hill, Grant No. T32-HS000032.

## Conflict of Interest

The authors declare that the research was conducted in the absence of any commercial or financial relationships that could be construed as a potential conflict of interest.

## Publisher’s Note

All claims expressed in this article are solely those of the authors and do not necessarily represent those of their affiliated organizations, or those of the publisher, the editors and the reviewers. Any product that may be evaluated in this article, or claim that may be made by its manufacturer, is not guaranteed or endorsed by the publisher.
